# Cryo-electron tomography reveals four-membrane architecture of the *Plasmodium* apicoplast

**DOI:** 10.1186/1475-2875-12-25

**Published:** 2013-01-19

**Authors:** Leandro Lemgruber, Mikhail Kudryashev, Chaitali Dekiwadia, David T Riglar, Jake Baum, Henning Stahlberg, Stuart A Ralph, Friedrich Frischknecht

**Affiliations:** 1 Parasitology, Department of Infectious Diseases, University of Heidelberg Medical School, Im Neuenheimer Feld 324, Heidelberg 69120, Germany; 2 Center for Cellular Imaging and Nano Analytics (C-CINA), Biozentrum, University of Basel, Mattenstrasse 26, Basel, CH-4058, Switzerland; 3 Department of Biochemistry and Molecular Biology, Bio21 Molecular Science and Biotechnology Institute, The University of Melbourne, Victoria 3010, Australia; 4 Division of Infection and Immunity, The Walter and Eliza Hall Institute of Medical Research, Parkville, VIC, 3052, Australia; 5 Department of Medical Biology, University of Melbourne, Parkville, VIC, 3052, Australia

**Keywords:** Apicoplast, Cryo-electron tomography, Symbiosis, Organelle interaction, Malaria

## Abstract

**Background:**

The apicoplast is a plastid organelle derived from a secondary endosymbiosis, containing biosynthetic pathways essential for the survival of apicomplexan parasites. The *Toxoplasma* apicoplast clearly possesses four membranes but in related *Plasmodium* spp. the apicoplast has variably been reported to have either three or four membranes.

**Methods:**

Cryo-electron tomography was employed to image merozoites of *Plasmodium falciparum* and *Plasmodium berghei* frozen in their near-native state. Three-dimensional reconstructions revealed the number of apicoplast membranes and the association of the apicoplast with other organelles. Routine transmission electron microscopy of parasites preserved by high-pressure freezing followed by freeze substitution techniques was also used to analyse apicoplast morphology.

**Results:**

Cryo-preserved parasites showed clearly four membranes surrounding the apicoplast. A wider gap between the second and third apicoplast membranes was frequently observed. The apicoplast was found in close proximity to the nucleus and to the rhoptries. The apicoplast matrix showed ribosome-sized particles and membranous whorls.

**Conclusions:**

The *Plasmodium* apicoplast possesses four membranes, as do the apicoplasts of other apicomplexan parasites. This is consistent with a four-membraned secondary endosymbiotic plastid ancestor.

## Background

All modern plastids are remnants of a primary endosymbiosis that occurred around one billion years ago, whereby a cyanobacterium was engulfed and retained by a eukaryote. The algae produced by this event possess plastids with two membranes corresponding to the double membranes of the gram-negative cyanobacteria. These algae later diverged into groups known today as Viridiplantae, Glaucophyta and Rhodophyta, and their plastids are known as primary endosymbionts. All existing plastids are either found in these algae, or in later eukaryotes that phagocytosed plastid-bearing organisms and retained their enclosed plastid [1]. Plastids acquired through capture of an organism that contained a primary endosymbiont are referred to as secondary endosymbionts. These organelles would initially have possessed four membranes, deriving from the two primary endosymbiont membranes, plus the plasma membrane of the primary endosymbiont and the phagosomal membrane of the engulfing eukaryote. In some lineages, one of those outer membranes has been secondarily lost to give rise to three-membrane plastids [2]. Algae with secondary endosymbionts can themselves be captured and retained (tertiary endosymbiosis) giving rise to plastids with more than four membranes [3].

Although all extant plastids are believed to derive from a single primary endosymbiosis event, secondary endosymbiosis has occurred a number of times, giving rise to multiple, unrelated plastid-bearing groups. One such hypothesized group is the chromalveolata, proposed to have arisen from a single secondary endosymbiosis involving a rhodophyte (red alga), that gave rise to extremely diverse organisms such as diatoms, dinoflagellates and apicomplexans [4].

The plastid of apicomplexan parasites - the apicoplast - has received considerable attention, in part because of the evolutionary implications of the presence of a plastid in this phylum [5,6], but mainly because apicomplexans are of immense medical and veterinary significance and the apicoplast is the target for important antiparasitic drugs [7]. If the chromalveolate hypothesis holds, the common ancestor of all apicomplexans possessed a four-membrane plastid, although at least one apicomplexan genus, *Cryptosporidium*, has subsequently lost its plastid. Apicoplasts lack a photosynthetic apparatus, but a recently identified close relative of the Apicomplexa, the alga *Chromera*[8], is photosynthetic and possesses a four-membrane plastid that appears to derive from the same red algal origin as the apicoplast [9].

Descriptions of apicoplast membranes are many and varied. Because the apicoplast identity was established long after apicomplexans had been ultrastructurally investigated there are many descriptions of the apicoplast with differing generic names, eg, spherical body, hohlzylinder and Golgi adjunct. Micrographs since the description of the apicoplast establish plastids with four membranes for many of the apicomplexans, including *Toxoplasma gondii*[10], *Sarcocystis*[11], *Garnia gonadati*[12], and *Babesia bovis*[13] as do many of the older micrographs for diverse apicomplexans (discussed in [14]). However, descriptions of the apicoplast of *Plasmodium* species, the causative agents of malaria, are conflicting. Some preparations appear to support a four-membrane interpretation in sporozoites [15]; however this was based on a single observation and thus was not further commented upon. In contrast other electron micrographs suggest an apicoplast with only three membranes [16-18]. This has led to an ongoing disagreement in the number of apicoplast membranes of *Plasmodium.* Loss of one of the four membranes clearly found in other apicomplexans would have considerable molecular consequences for understanding protein trafficking and biogenesis for the *Plasmodium* apicoplast, so the resolution of this question is desirable.

To investigate this discrepancy, electron microscopy of cryopreserved parasites from *Plasmodium falciparum* and *Plasmodium berghei* coupled to tomographic reconstructions was used. Cryo-electron tomography is widely regarded to introduce the fewest artefacts during preparation as the specimen is rapidly frozen (within a few milliseconds) thus preserving molecular details and membrane arrangements [19,20]. This technique has been used successfully to investigate membranous and cytoskeletal structures in sporozoites of *P. berghei*[15,21-23] and Maurer’s clefts of *P. falciparum* infected red blood cells [24,25]. Cryo-electron tomography does not include staining with heavy metal salts and thus provides lower contrast, though being sufficient to examine membranes [26]. Reconstructed tomograms of both *P. falciparum* and *P. berghei* merozoites clearly show the apicoplast with four delimiting membranes. These membranes often appear paired with a gap between the second and third membranes. In some individual sections through the tomograms, only three membranes are apparent around some apicoplasts, but three-dimensional reconstructions of these resolves the local appositions of two membranes to a total of four delimiting membranes.

## Methods

### Ethics statement

All animal experiments were performed according to the FELASA and GV-SOLAS standard guidelines. Animal experiments were approved by the German authorities (Regierungspräsidium Karlsruhe).

### Obtaining *Plasmodium* merozoites

*Plasmodium falciparum* parasites from strains 3D7 and D10 were maintained using standard procedures. Cultures were grown in human erythrocytes in RPMI 1640 supplemented with L-glutamine, HEPES, hypoxanthine, and gentamycin. Some cultures were supplemented with human heat-inactivated serum and albumax, and others were supplemented with albumax alone. Parasites were incubated at 37°C in a gas mixture of 5% CO_2_, 1% O_2_, and 94% N_2_. For merozoites subject to cryopreservation prior to electron tomography, late schizonts were harvested by magnetic cell sorting and resuspended in medium. Merozoites were mechanically isolated by passage in a needle.

For *P. falciparum* merozoites to be subject to conventional glutaraldehyde and osmium fixation or for high pressure freezing and freeze substitution (see below), purification was performed as described by Boyle and colleagues [27].

To obtain *P. berghei* merozoites, blood of an infected mouse was collected in T-medium (RPMI, 20% FCS, 0.03% gentamicin) plus heparin, centrifuged (110 × *g* for 8 min) and resuspended in T-medium and placed on a shaker (50 rpm) overnight at 37°C. Mature schizonts were harvested with a Nycodenz gradient (Axis-Shield PoC, Norway), washed and resuspended in RPMI medium prior to freezing.

### Cryo-electron tomography

Tomography was performed essentially as described before [15,21,23,24,28]. Merozoites in RPMI medium mixed with 10 nm colloidal gold particles were transferred onto glow-discharged holey carbon Quantifoil EM grids. Grids were blotted at 90% humidity using a Vitrobot (FEI). After removing the liquid excess, the material was rapidly plunge frozen into liquid ethane and stored in liquid nitrogen. The grids were observed in a Titan Krios (FEI) operating at 300 kV and equipped with a Gatan post column energy filter and post-GIF CCDs operating at liquid nitrogen temperature. Tilt series were acquired with an increment of 2º covering -60º to +60º, with a cumulative dose under 10,000 electrons/nm^2^ and a defocus of -3 to -15 μm on a 2k Ultrascan 1000 CCD camera (Gatan).

For this study a total of 12 (for *P. berghei*) and 24 (for *P. falciparum*) tomograms were reconstructed by weighted back-projection using the Etomo program in the IMOD software package (Boulder Laboratory for 3D electron microscopy) [29]. Reconstructed tomograms were filtered using non-linear anisotropic diffusion [30]. Visualization, volume rendering and segmentation were performed using the 3dmod program of the IMOD package [29].

### High-pressure freezing and freeze substitution

Purified *P. falciparum* merozoites from strain D10 were allowed to invade red blood cells, then 10 min post invasion were high-pressure frozen using a LeicaEM high-pressure freezer. The infected erythrocyte samples were freeze substituted with 1% uranyl acetate at -90°C for 24 hrs using a Leica AFS automatic freeze substitution machine. Samples were further freeze substituted with acetone and then infiltrated with increasing concentrations of Lowicryl HM20 resin in acetone. Resin was polymerized using UV light treatment for 48 hrs at -45°C then a further 48 hrs at room temperature. Sections of approximately 90 nanometers were cut at room temperature, stained with uranyl acetate and lead citrate and examined using a Philips CM120 BioTWIN transmission electron microscope at 120 kV (Advanced Microscopy facility, School of Botany, the University of Melbourne, Australia).

### Conventional transmission electron microscopy

Purified *P. falciparum* merozoites (as described above) were fixed in 2.5% glutaraldehyde in pH 7.4 phosphate buffer on ice. Cells were washed in phosphate buffer and fixed in 1% osmium tetroxide (ProSciTech, Australia) in 0.1 M phosphate buffer for one hour on ice. Samples were washed in water, then dehydrated in increasing concentrations of ethanol before embedding in LR Gold resin (ProSciTech, Australia) and polymerized using benzoyl peroxide (SPI-Chem, USA). Sections were cut, stained and inspected as described above.

## Results and discussion

**Figure 1:**
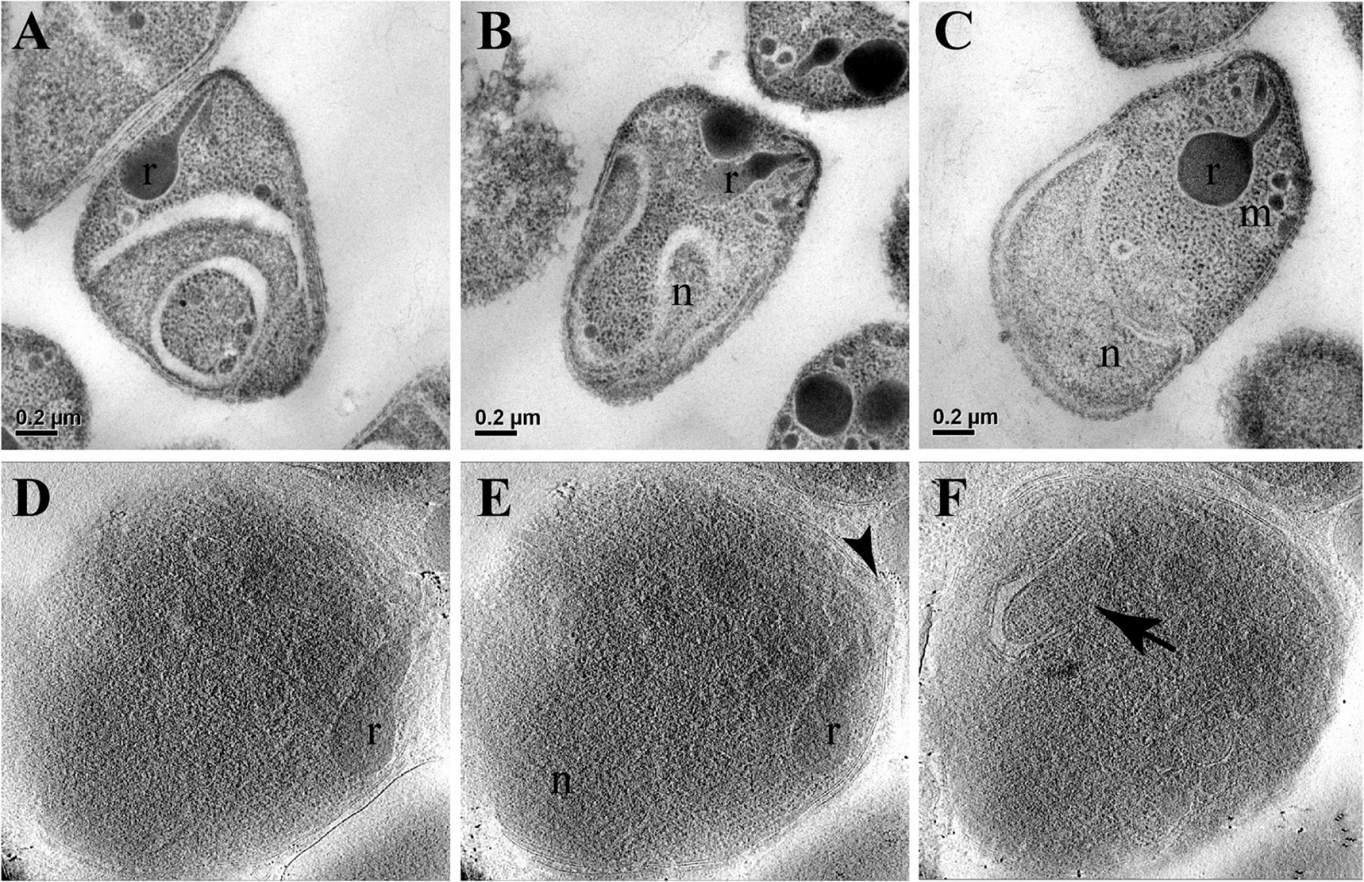
**Structural organization of merozoites processed by different electron microscopy techniques. A** to **C **– different examples of merozoites processed by conventional transmission electron microscopy. Rhoptries (r), micronemes (m) and the nucleus (n) are clearly observed. **D **to **F – **different sections from a tomogram of a cryo-preserved merozoite. Although more difficult to identify, some structures stand out like the rhoptries (r), the polar rings (arrowhead) and the apicoplast (arrow).

In images of merozoites processed in conventional transmission electron microscopy protocols, some organelles are clearly observed such as rhoptries, while even the nucleus can appear in a distorted fashion (Figures 1A-C). This is due to the use of fixative compounds, dehydration and embedding in resin. Better preservation of structural organization can be achieved when specimens are frozen at high pressure and all the steps for transmission electron microscopy are carried out at low temperatures [31]. But even then, where the post-fixation processing is carried out in low temperatures, high-pressure freezing still requires the use of fixative, dehydration and resin, which in turn can also lead to modifications in the structural organization of the merozoites. In addition, the material is stained and uncontrolled, selective depositions of heavy metal salts further obscure fine details just as in classic transmission electron microscopy. Thus, these techniques can lead to misinterpretation. These considerations are not a concern in cryo-electron microscopy, where the parasites are rapidly frozen close to their native state and not subjected to any other procedure that may affect fine structure [20]. However, images of merozoites processed for cryo-electron microscopy, appear more noisy (Figures 1D-F) and it can be more difficult to readily identify the organelles.

**Figure 2:**
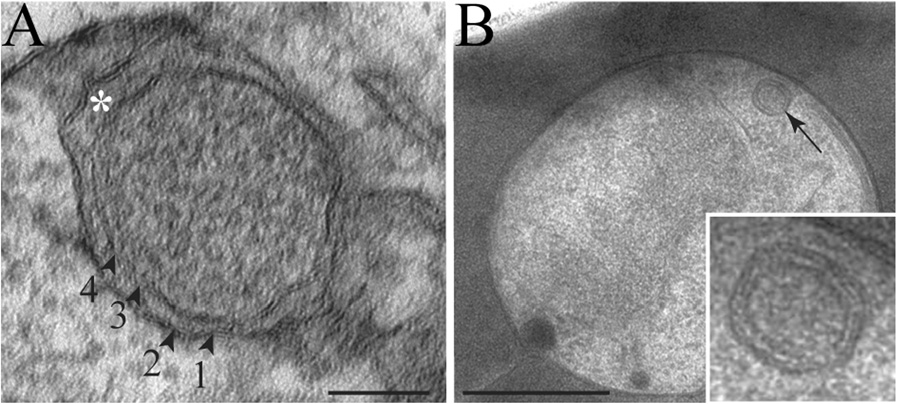
**Apicoplast ultrastructure in *Plasmodium falciparum *merozoites. A **– Apicoplast of a *Plasmodium falciparum *merozoite processed by glutaraldehyde and osmium chemical fixation and examined by transmission electron microscopy. The plastid is clearly surrounded by four membranes (arrowheads), with a gap between the two outermost membranes and the two innermost membranes (asterisk) in some regions. **B** – When processed by high-pressure freezing and freeze substitution, the apicoplast (arrow) ultrastructure was better preserved, also presenting four membranes. The gap between the membranes 2 and 3 is still present. Scale bars: A = 50 nm; B = 200 nm.

Merozoites processed in conventional transmission electron microscopy protocols, ie, room temperature processing, appear to show apicoplasts with three [16,17] or four apparent membranes (Figure 2A). High-pressure frozen merozoites presented four membranes delimiting the apicoplast (Figure 2B). Both in routine (Figure 2A) and in high-pressure (Figure 2B) processing, the apicoplast presented a granular matrix with the first and the second membranes as well as the third and the fourth close to each other. Also, a gap between the second and the third membranes was observed. This general organization is similar to that described previously for the *Toxoplasma gondii* apicoplast [32].

**Figure 3:**
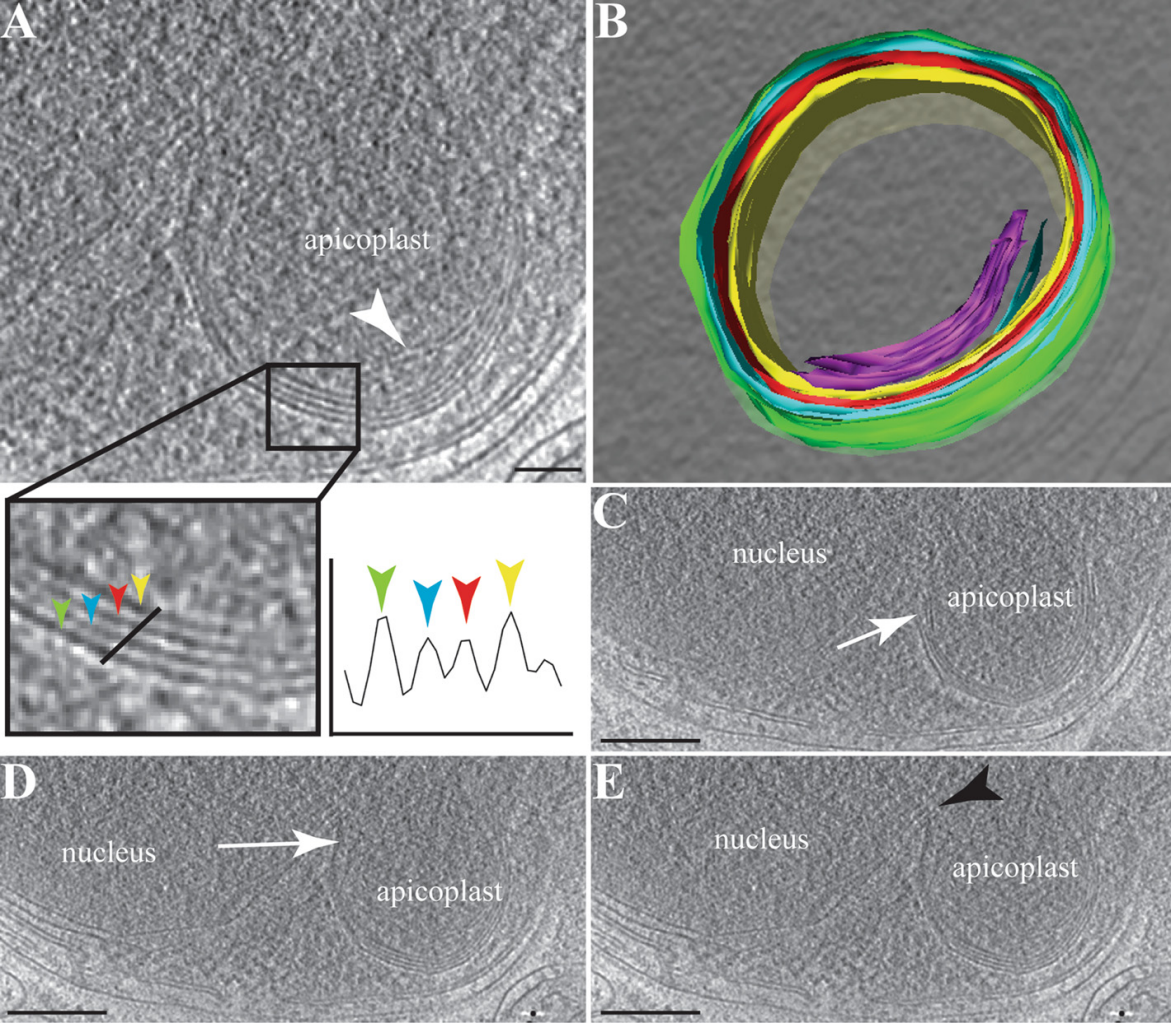
**Tomographic reconstruction of the apicoplast in a *Plasmodium berghei *merozoite. **Panel **A **shows a virtual section of an apicoplast, with the four apicoplast membranes indicated by the coloured arrowheads and a graphed density distribution. The apicoplast matrix presents an internal membranous whorl as indicated (white arrowhead). Panel **B **shows a rendered model of the apicoplast, highlighting the internal whorls (purple) and the four membranes (yellow, red, blue and green). Panels **C** and **D **show sections where a gap is observed between membranes 2 and 3 (white arrows). In Panel **E**, a close apposition is observed between the nucleus and the apicoplast (black arrowhead). Scale bars: A = 60 nm; B-E = 20 nm.

**Figure 4:**
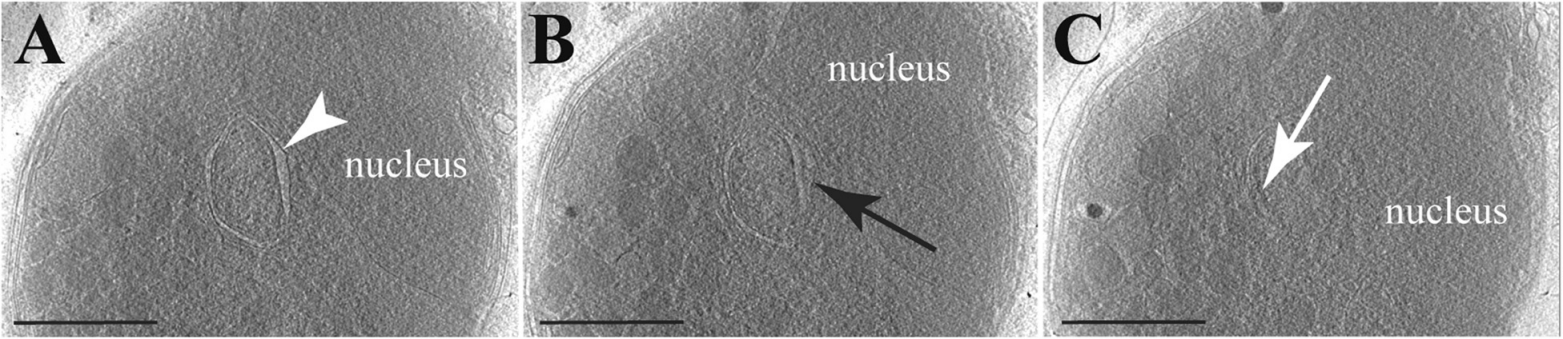
**Gaps between apicoplast membranes. A **to **C – **Different sections of a reconstructed tomogram of a *Plasmodium berghei *merozoite. The apicoplast with its four membranes is located close to the nucleus. The plastid presents a widening between the two inner membranes and the two outer membranes (white arrowhead). A gap between the two outermost membranes is also occasionally observed (black arrow). In **C**, electron dense structures (white arrow) are present on some apicoplast membranes, possibly corresponding to protein complexes such as import apparatus. Scale bar = 50 nm.

Reconstructed tomograms of cryo-fixed *P. berghei* merozoites again showed four membranes delimiting the apicoplast (Figures 3 and 4). This is quantitatively observed on the density profiles across an apicoplast, revealing a clear peak for each of the four membranes (Figure 3A) and in a 3D model of the organelle (Figure 3B and Additional file 1: Video 1). It is noteworthy that membranous profiles were also observed within the apicoplast matrix (Figures 3A and 3B). The biological nature and purpose of these matrix membranes is unclear, but similar structures have previously been described inside the apicoplast of *Plasmodium*[16] and *Toxoplasma*[32]. Cryo-electron tomography revealed gaps not only between the second and the third membranes (Figures 3C and 4A) but also, though less frequently, between the third and the fourth membranes (Figure 3D) and the first and the second membranes (Figure 4B).

**Figure 5:**
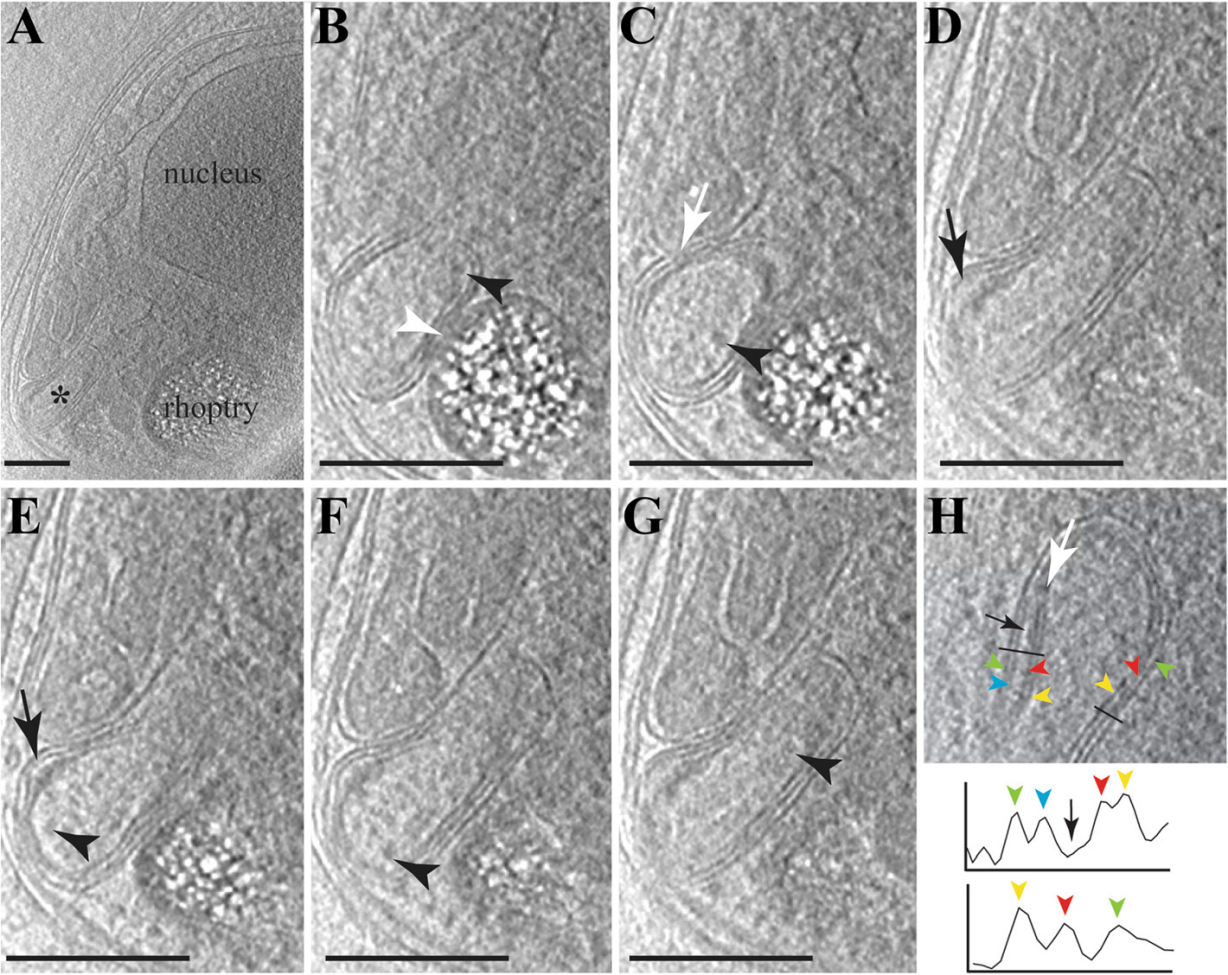
**Slices through a reconstructed tomogram of a *Plasmodium falciparum *merozoite. **The apicoplast appears close to the nucleus and the rhoptries. White arrowhead points to an interaction between the apicoplast membrane and the rhoptry membrane. Within the apicoplast matrix, several ribosome-like particles were observed (black arrowheads). Black arrows indicate the different regions with a widening between the two outermost membranes and the two innermost membranes. White arrows indicate electron dense structures (possibly translocons) in the apicoplast membranes. In **H**, the density distribution over the lines show that the apicoplast has four membranes (top profile), but in some slices through the tomogram only three electron dense lines are apparent (bottom profile), due to the close apposition of two membranes. Scale bars: A-G = 25 nm.

The apicoplast of *P. falciparum* merozoites (Figure 5) showed the same general morphology as those of *P. berghei*. The apicoplast was not only located close to the nucleus but its outer most membrane was also observed in close apposition to the rhoptries and the mitochondrion (Figures 5B-G). Gaps between the membranes were also observed (Figures 5D and E). Ribosome-like particles were clearly observed in the apicoplast’s matrix (Figures 5C and 5E-G). Also, large protein complexes were present within the gap between the membranes (Figure 5B). However the high noise in tomograms due to the thickness of the reconstructed volumes (600–800 nm) hampered further interpretation of these complexes. Similar gaps and material between membranes was also observed in *Eimeria*[33].

The relationship between the apicoplast and the endoplasmic reticulum (ER) has been the subject of some conjecture [16,31,34]. In heterokonts the plastid resides within the lumen of the ER [35], and a similar arrangement is formally possible for the apicoplast (see reviews in [14,34,36] for more detailed discussions of such models). Such an arrangement might predict that the outer membrane of the plastid contained ribosomes, necessary for the insertion of apicoplast-targeted proteins. As with previous examinations, such ribosomes on the outer membrane of the plastid, and continuities between the membranes of the plastid and ER were not observed. However, in merozoites, where all organelles are more closely apposed, the apicoplast was observed in close proximity to the nucleus and ER (Figures 3E and 4A), as previously described for *Plasmodium*[16], *Toxoplasma*[32] and *Sarcocystis*[11]. Although there appeared to be some contact points with the nuclear envelope and with the ER (Figure 3E), no continuity between these membranes is apparent.

Much has been discussed about how proteins are transported across the apicoplast’s surrounding membranes, with various membranous translocon proteins already described [37-41]. In the acquired cryo-tomograms, structures were observed in merozoites’ apicoplast of both *P. berghei* and *P. falciparum*, potentially resembling a translocon (Figures 4C and 5C), although this cannot be resolved with the current images. These features were located between the second and the third membranes.

Going through the virtual sections of a reconstructed tomogram, one can observe that depending on the section the apicoplast can show three or four membranes, or even at the same section both arrangements (Figure 5H). This is usually due to the close apposition of the second membrane with the first one, which can explain the discrepancies in the previously published apicoplast structure descriptions [16-18].

## Conclusion

The *Plasmodium* apicoplast was shown to contain four membranes as in other apicomplexan parasites. The apicoplast was found in close proximity to the membranes of the nucleus, inner membrane complex, the mitochondrion and rhoptries. The apicoplast membranes are arranged such that the outer and innermost membranes were closely associated to each other while some space occurred between the two doublet membranes. The presented data reconciles molecular evidence as to how transport into the apicoplast is thought to work in *Toxoplasma* and *Plasmodium* with electron microscopy data and shows the capacity of cryo-electron tomography to reveal fine structural details in thin parasites.

## Competing interests

The authors declare that they have no competing interests.

## Authors’ contribution

LL, MK, SAR, FF designed the experiments and carried out data analysis; LL, MK, CD, DTR performed the experiments; HS, JB contributed essential materials; LL, SAR, FF wrote the paper. All authors read and approved the final manuscript.

## Supplementary Material

Additional file 1 **Video 1. **View of the 3D model from a reconstructed tomogram of a *Plasmodium berghei *apicoplast. This video is related to Figure 3. Click here for file

## References

[B1] Gould SB Waller RF McFadden GI Plastid evolution Annu Rev Plant Biol 2008 59 491 517 10.1146/annurev.arplant.59.032607.092915 18315522

[B2] Dodge J A survey of chloroplast ultrastructure in the Dinophyceae Phycologia 1975 14 253 363 10.2216/i0031-8884-14-4-253.1

[B3] Archibald JM The puzzle of plastid evolution Curr Biol 2009 19 R81 R88 10.1016/j.cub.2008.11.067 19174147

[B4] Cavalier-Smith T Principles of protein and lipid targeting in secondary symbiogenesis: euglenoid, dinoflagellate, and sporozoan plastid origins and the eukaryote family tree J Eukaryot Microbiol 1999 46 347 366 10.1111/j.1550-7408.1999.tb04614.x 18092388

[B5] McFadden GI Reith ME Munholland J Lang-Unnasch N Plastid in human parasites Nature 1996 381 482 10.1038/381482a0 8632819

[B6] Fast NM Kissinger JC Roos DS Keeling PJ Nuclear-encoded, plastid-targeted genes suggest a single common origin for apicomplexan and dinoflagellate plastids Mol Biol Evol 2001 18 418 426 10.1093/oxfordjournals.molbev.a003818 11230543

[B7] Ralph SA Van Dooren GG Waller RF Crawford MJ Fraunholz MJ Foth BJ Tonkin CJ Roos DS McFadden GI Metabolic maps and functions of the *Plasmodium falciparum* apicoplast Nat Rev Microbiol 2004 2 203 216 10.1038/nrmicro843 15083156

[B8] Moore RB Oborník M Janouskovec J Chrudimský T Vancová M Green DH Wright SW Davies NW Bolch CJS Heimann K Slapeta J Hoegh-Guldberg O Logsdon JM Carter DA A photosynthetic alveolate closely related to apicomplexan parasites Nature 2008 451 959 963 10.1038/nature06635 18288187

[B9] Janouskovec J Horák A Oborník M Lukes J Keeling PJ A common red algal origin of the apicomplexan, dinoflagellate, and heterokont plastids Proc Natl Acad Sci USA 2010 107 10949 10954 10.1073/pnas.1003335107 20534454 PMC2890776

[B10] Kohler S Delwiche CF Denny PW Tilney LG Webster P Wilson R Palmer JD Roos DS A plastid of probable green algal origin in apicomplexan parasites Science 1997 275 1485 1489 10.1126/science.275.5305.1485 9045615

[B11] Tomova C Geerts WJC Müller-Reichert T Entzeroth R Humbel BM New comprehension of the apicoplast of *Sarcocystis* by transmission electron tomography Biol Cell 2006 98 535 545 10.1042/BC20060028 16706752

[B12] Diniz JAP Silva EO Lainson R De Souza W The fine structure of *Garnia gonadati* and its association with the host cell Parasitol Res 2000 86 971 977 10.1007/PL00008528 11133112

[B13] Caballero MC Pedroni MJ Palmer GH Suarez CE Davitt C Lau AOT Characterization of acyl carrier protein and LytB in *Babesia bovis* apicoplast Mol Biochem Parasitol 2012 181 125 133 10.1016/j.molbiopara.2011.10.009 22057350 PMC3278595

[B14] Waller RF McFadden GI The apicoplast: a review of the derived plastid of apicomplexan parasites Curr Issues Mol Biol 2005 7 57 79 15580780

[B15] Kudryashev M Lepper S Stanway R Bohn S Baumeister W Cyrklaff M Frischknecht F Positioning of large organelles by a membrane associated cytoskeleton in *Plasmodium* sporozoites Cell Microbiol 2010 12 362 371 10.1111/j.1462-5822.2009.01399.x 19863555

[B16] Hopkins J Fowler R Krishna S Wilson I Mitchell G Bannister L The plastid in *Plasmodium falciparum* asexual blood stages: a three-dimensional ultrastructural analysis Protist 1999 150 283 295 10.1016/S1434-4610(99)70030-1 10575701

[B17] Bannister LH Hopkins JM Fowler RE Krishna S Mitchell GH A brief illustrated guide to the ultrastructure of *Plasmodium falciparum* asexual blood stages Parasitol Today 2000 16 427 433 10.1016/S0169-4758(00)01755-5 11006474

[B18] Bannister L Margos G Hopkins JM Making a home for Plasmodium post-genomics: ultrastructural organization of the blood stages 2005 Washington D.C: Molecular Approaches to Malaria. ASM Press

[B19] Lepper S Merkel M Sartori A Cyrklaff M Frischknecht F Rapid quantification of the effects of blotting for correlation of light and cryo-light microscopy images J Microsc 2010 238 21 26 10.1111/j.1365-2818.2009.03327.x 20384834

[B20] Lučić V Förster F Baumeister W Structural studies by electron tomography: from cells to molecules Annu Rev Biochem 2005 74 833 865 10.1146/annurev.biochem.73.011303.074112 15952904

[B21] Cyrklaff M Kudryashev M Leis A Leonard K Baumeister W Ménard R Meissner M Frischknecht F Cryoelectron tomography reveals periodic material at the inner side of subpellicular microtubules in apicomplexan parasites J Exp Med 2007 204 1281 1287 10.1084/jem.20062405 17562819 PMC2118598

[B22] Kudryashev M Lepper S Baumeister W Cyrklaff M Frischknecht F Geometric constrains for detecting short actin filaments by cryogenic electron tomography PMC Biophys 2010 3 6 10.1186/1757-5036-3-6 20214767 PMC2844354

[B23] Kudryashev M Münter S Lemgruber L Montagna G Stahlberg H Matuschewski K Meissner M Cyrklaff M Frischknecht F Structural basis for chirality and directional motility of *Plasmodium* sporozoites Cell Microbiol 2012 14 1757 1768 10.1111/j.1462-5822.2012.01836.x 22776715 PMC4116596

[B24] Henrich P Kilian N Lanzer M Cyrklaff M 3-D analysis of the *Plasmodium falciparum* Maurer’s clefts using different electron tomographic approaches Biotechnol J 2009 4 888 894 10.1002/biot.200900058 19492330

[B25] Cyrklaff M Sanchez CP Kilian N Bisseye C Simpore J Frischknecht F Lanzer M Hemoglobins S and C Interfere with Actin Remodeling in *Plasmodium falciparum*-Infected Erythrocytes Science 2011 334 1283 1286 10.1126/science.1213775 22075726

[B26] Leforestier A Lemercier N Livolant F Contribution of cryoelectron microscopy of vitreous sections to the understanding of biological membrane structure Proc Natl Acad Sci USA 2012 109 8959 8964 10.1073/pnas.1200881109 22615384 PMC3384177

[B27] Boyle MJ Wilson DW Richards JS Riglar DT Tetteh KKA Conway DJ Ralph SA Baum J Beeson JG Isolation of viable *Plasmodium falciparum* merozoites to define erythrocyte invasion events and advance vaccine and drug development Proc Natl Acad Sci USA 2010 107 14378 14383 10.1073/pnas.1009198107 20660744 PMC2922570

[B28] Kühni-Boghenbor K Ma M Lemgruber L Cyrklaff M Frischknecht F Gaschen V Stoffel M Baumgartner M Actin-mediated plasma membrane plasticity of the intracellular parasite *Theileria annulata* Cell Microbiol 2012 14 1867 1879 10.1111/cmi.12006 22891986

[B29] Kremer JR Mastronarde DN McIntosh JR Computer visualization of three-dimensional image data using IMOD J Struct Biol 1996 116 71 76 10.1006/jsbi.1996.0013 8742726

[B30] Frangakis AS Hegerl R Noise reduction in electron tomographic reconstructions using nonlinear anisotropic diffusion J Struct Biol 2001 135 239 250 10.1006/jsbi.2001.4406 11722164

[B31] Studer D Humbel BM Chiquet M Electron microscopy of high pressure frozen samples: bridging the gap between cellular ultrastructure and atomic resolution Histochem Cell Biol 2008 130 877 889 10.1007/s00418-008-0500-1 18795316

[B32] Tomova C Humbel BM Geerts WJC Entzeroth R Holthuis JCM Verkleij AJ Membrane contact sites between apicoplast and ER in *Toxoplasma gondii* revealed by electron tomography Traffic 2009 10 1471 1480 10.1111/j.1600-0854.2009.00954.x 19602198

[B33] Ferguson DJ Campbell SA Henriquez FL Phan L Mui E Richards TA Muench SP Allary M Lu JZ Prigge ST Tomley F Shirley MW Rice DW McLeod R Roberts CW Enzymes of type II fatty acid synthesis and apicoplast differentiation and division in *Eimeria tenella* Int J Parasitol 2007 37 33 51 10.1016/j.ijpara.2006.10.003 17112527 PMC2803676

[B34] Tonkin CJ Kalanon M McFadden GI Protein targeting to the malaria parasite plastid Traffic 2008 9 166 175 17900270 10.1111/j.1600-0854.2007.00660.x

[B35] Gibbs SP The route of entry of cytoplasmically synthesized proteins into chloroplasts of algae possessing chloroplast ER J Cell Sci 1979 35 253 266 422674 10.1242/jcs.35.1.253

[B36] Roos DS Crawford MJ Donald RG Kissinger JC Klimczak LJ Striepen B Origin, targeting, and function of the apicomplexan plastid Curr Opin Microbiol 1999 2 426 432 10.1016/S1369-5274(99)80075-7 10458993

[B37] Kalanon M Tonkin CJ McFadden GI Characterization of two putative protein translocation components in the apicoplast of *Plasmodium falciparum* Eukaryot Cell 2009 8 1146 1154 10.1128/EC.00061-09 19502580 PMC2725556

[B38] Van Dooren GG Tomova C Agrawal S Humbel BM Striepen B *Toxoplasma gondii* Tic20 is essential for apicoplast protein import Proc Natl Acad Sci USA 2008 105 13574 13579 10.1073/pnas.0803862105 18757752 PMC2533231

[B39] Brooks CF Johnsen H Van Dooren GG Muthalagi M Lin SS Bohne W Fischer K Striepen B The *Toxoplasma* apicoplast phosphate translocator links cytosolic and apicoplast metabolism and is essential for parasite survival Cell Host Microbe 2010 7 62 73 10.1016/j.chom.2009.12.002 20036630 PMC3013619

[B40] Karnataki A DeRocher A Coppens I Nash C Feagin JE Parsons M Cell cycle-regulated vesicular trafficking of *Toxoplasma* APT1, a protein localized to multiple apicoplast membranes Mol Microbiol 2007 63 1653 1668 10.1111/j.1365-2958.2007.05619.x 17367386

[B41] Glaser S Higgins MK Overproduction, purification and crystallization of PfTic22, a component of the import apparatus from the apicoplast of *Plasmodium falciparum* Acta Crystallogr Sect F Struct Biol Cryst Commun 2012 68 351 354 10.1107/S1744309112004952 22442242 PMC3310550

